# A retrospective assessment of the implementation of virtual medical physics educational initiatives using the consolidated framework for implementation research

**DOI:** 10.3389/fmed.2025.1625455

**Published:** 2025-07-29

**Authors:** Noelle Fedor, E. Axel Abrahamson, Joseph Weygand

**Affiliations:** Division of Global Radiation Oncology, Department of Radiation Oncology and Applied Sciences, Dartmouth-Hitchcock Medical Center, Lebanon, NH, United States

**Keywords:** global health, medical physics, implementation sceince, education, training

## Abstract

Global disparities in access to radiation medicine are driven not only by infrastructure deficits but also by shortages of trained medical physicists. To address these gaps, RAD-AID International implemented four virtual educational initiatives in Kenya, Guyana, and Türkiye, along with a global lecture series, each aimed at strengthening local capacity in medical physics. Implementing such programs across diverse low- and middle-income country (LMIC) contexts presents significant challenges, particularly with regard to adaptability, engagement, and sustainability. To evaluate these efforts, we applied the Consolidated Framework for Implementation Research (CFIR) and the CFIR Expert Recommendations for Implementing Change (ERIC) Matching Tool to retrospectively identify barriers and recommend strategies to strengthen future initiatives. Stakeholders across all programs consistently cited limited access to knowledge and information as a key barrier. In response, the ERIC strategy “Conduct Educational Meetings” was highly endorsed, with 79% of experts identifying it as a top recommendation. Additional barriers, such as adaptability, planning, and responsiveness to patient needs, were matched with strategies including promoting adaptability, developing formal implementation blueprints, and engaging patients and families. By linking CFIR constructs to concrete examples, this study demonstrates the utility of structured implementation frameworks in radiological education and underscores the need for contextual sensitivity in resource-limited settings.

## Introduction

Radiation medicine is fundamental to modern cancer care; however, access to radiotherapy remains profoundly unequal. In LMICs, over 90% of the population lacks access to basic radiotherapy services (1). Radiological imaging is similarly essential, supporting cancer detection, staging, treatment planning, and disease monitoring, with an estimated 3.2% of global cancer deaths attributable to insufficient imaging access (2). The global cancer burden is expected to increase by 77% from 2022 to 2050, disproportionately affecting LMICs and placing increasing pressure on diagnostic and therapeutic infrastructure (3). Radiological services such as computed tomography (CT), magnetic resonance imaging (MRI), and nuclear medicine also support the diagnosis and management of cardiovascular disease, tuberculosis, and maternal complications. However, disparities remain stark: LMICs often have fewer than one CT scanner per million people compared to nearly 40 per million in high-income countries (4). In recognition of this, the World Health Assembly recently issued a resolution calling for the global expansion of diagnostic imaging capacity, emphasizing workforce development as a central pillar (5).

These disparities are not solely infrastructural but also reflect critical workforce shortages. Medical physicists play an essential role in ensuring the safe and effective delivery of radiological care, yet many LMICs lack accredited training programs altogether. This has led to underutilized equipment and acute shortages of qualified personnel (23). Despite their importance, medical physicists are often overlooked in global health initiatives, which tend to focus more broadly on service delivery or infrastructure expansion. In addition, continuing education opportunities in medical subspecialties also remain fragmented or inaccessible in many LMICs (6).

To help address these gaps, RAD-AID International, a non-profit organization that works to improve access to medical imaging and radiology services (21), launched four virtual educational initiatives. These initiatives included the following: (1) a global medical physics education lecture series; (2) a diagnostic and therapeutic physics course for radiation oncology residents in Kenya; (3) a medical imaging physics course for radiology residents in Guyana; and (4) a comprehensive MRI course in Türkiye. The Global Medical Physics Education Lecture Series (GMPELS), delivered between 2022 and 2024, enrolled 226 participants, more than half of whom were practicing clinical medical physicists. The Guyana initiative, established in 2021 at Georgetown Public Hospital, was designed for radiology residents with limited prior exposure to physics (7). In Kenya, the program supported 26 residents at the University of Nairobi and aligned with national efforts aimed at staffing 12 new radiotherapy centers (8). The Turkish MRI course introduced 160 learners to foundational and advanced imaging principles over 9 weeks. Each initiative was tailored to meet local needs but differed in delivery format, audience, and content focus. Table 1 summarizes the design and characteristics of each program.

**Table 1:** An overview of virtual medical physics initiatives sponsored by RAD-AID.

Program	Location	Audience	Number of participants	Duration	Focus
Kenya	University of Nairobi	Radiation oncology residents	26	1 year	Introduction to diagnostic and therapeutic medical physics, covering radiation production and detection, imaging modalities (X-ray, CT, MRI, ultrasound, nuclear medicine), radiation safety, treatment planning, brachytherapy, quality assurance, and advanced techniques, such as IMRT and stereotactic procedures
Guyana	University of Georgetown	Radiology residents with limited physics background	4	5 weeks	Medical imaging modalities (X-ray, CT, MRI, ultrasound, fluoroscopy, mammography, and nuclear medicine) with a focus on basic physics, image quality, safety, and informatics
Türkiye MRI Course	Türkiye	General medical imaging learners(??)	160	9 weeks	Introduction to medical imaging principles (NMR, spatial encoding, agentic relaxation, sequence design, quality assurance, MRI safety)
Global Medical Physics Education Lecture Series (GMPELS)	Virtual	Medical physicists, medical physics students, and individuals engaged in academic medical physics work	226	Ongoing monthly presentations	A mix of radiotherapy and diagnostic imaging topics, including ultrasound and MRI physics, brachytherapy, modern linear accelerator and proton therapy techniques, treatment planning, dosimetry, imaging modalities, motion management, surface guidance, safety, and anatomy

This table provides a comparative overview of each course design, each tailored to specific learner needs and regional contexts.

These initiatives reflect a broader shift toward technology-mediated health education as a means of improving professional capacity at scale. Online platforms offer cost-effective and flexible mechanisms for delivering specialized content across geographic and resource constraints. One illustrative example is Brazil’s Virtual Learning Environment of the Unified Health System (AVASUS), which is among the largest public health education platforms in the world. With more than 2 million registered learners, AVASUS has demonstrated success in expanding access to continuing professional development and improving readiness for health system service delivery (9). Similar digital platforms have been shown to improve health workforce performance, particularly when tailored to local needs and paired with sustained engagement strategies (20).

Despite these advantages, virtual education initiatives face meaningful implementation challenges. These challenges include difficulties in adapting content to local clinical contexts, engaging participants over time, and ensuring sustainability beyond the initial training period. Programs that span diverse LMIC settings must also contend with variability in infrastructure, staffing, institutional priorities, and learner needs (10–12). Understanding how these factors influence program outcomes is essential for optimizing educational investments.

To evaluate these dimensions, we applied the Consolidated Framework for Implementation Research (CFIR), a comprehensive implementation science model that identifies barriers and facilitators across five domains: intervention characteristics, outer setting, inner setting, characteristics of individuals, and implementation process (13, 19). In addition, we used the CFIR Expert Recommendations for Implementing Change (ERIC) Matching Tool, which links each identified barrier to expert-endorsed implementation strategies based on established consensus (14–16). The matching tool enables users to retrospectively evaluate implementation efforts and identify strategies likely to improve adoption, fidelity, and sustainability.

This study aims to assess the implementation of RAD-AID’s four educational programs using CFIR and the ERIC Matching Tool. Specifically, we sought to answer two research questions: (1) What implementation barriers were encountered across the four programs? and (2) Which ERIC strategies were most frequently matched to these barriers? We hypothesized that, although challenges would differ across programs, certain strategies would emerge as broadly applicable and that linking CFIR constructs to recommended actions would provide insight into strengthening future initiatives.

## Methods

To evaluate the implementation of four educational initiatives, we administered a structured questionnaire to stakeholders involved in the design and delivery of each program. The questionnaire was organized by the five domains of the Consolidated Framework for Implementation Research (CFIR) and asked respondents to select two perceived barriers from each domain. This yielded a total of 10 barrier selections per stakeholder. Participants included instructors, evaluators, and course organizers from Kenya (*n* = 3), Guyana (*n* = 3), Türkiye (*n* = 4), and the Global Medical Physics Education Lecture Series (GMPELS; *n* = 2). Although the total sample size was small, it reflected complete participation from all individuals directly engaged in each program and represented the complete set of available implementation stakeholders.

Once selected, CFIR barriers were entered into the CFIR Expert Recommendations for Implementing Change (ERIC) Matching Tool (14). This tool links each barrier to a list of 73 evidence-based implementation strategies developed through a consensus process involving 169 researchers, practitioners, and administrators. These experts identified and ranked the strategies they believed were most effective for overcoming specific CFIR-defined barriers (15, 16). For each queried barrier, the matching tool provides a list of strategies accompanied by the percentage of experts who endorsed each one among their top seven choices.

Barrier identification and strategy matching were conducted separately for each of the four programs. To interpret the outputs, we applied two complementary approaches. First, we calculated the cumulative percentage of expert endorsements for each strategy across all barriers within a program, which allowed us to identify those strategies most broadly applicable. Second, we highlighted the highest-ranked strategy for each specific barrier, enabling a more context-sensitive understanding of implementation needs. Because the study relied on a structured selection of pre-defined CFIR constructs rather than open-ended qualitative responses, thematic coding and inter-rater validation were not applied. The focus of the analysis was not on generating emergent themes but on mapping stakeholder-selected barriers to existing constructs in the CFIR framework and linking them to implementation strategies. All data were processed using Microsoft Excel, and no additional analytic software was used. De-identified questionnaire responses, CFIR-ERIC Matching Tool outputs, and related supporting materials are available from the corresponding author upon request.

## Results

Table 2 summarizes the distribution of identified barriers by stakeholders across the five CFIR domains for each educational program. The domain-level percentages reflect the extent to which barriers in each domain were perceived by stakeholders as relevant to implementation. The Outer Setting domain accounted for the highest proportion of barriers in Türkiye (100%) and Kenya (75%), suggesting that external influences were dominant challenges in these contexts. Characteristics of Individuals were most prominent in Guyana (75%) and Türkiye (75%), reflecting challenges related to *Individual Knowledge and Beliefs*, or *Readiness for Implementation*. Barriers within the Inner Setting domain were less frequently selected across all programs, ranging from 21% (GMPELS) to 36% (Kenya), while *Process* barriers were moderately represented across all programs.

**Table 2:** Distribution of selected barriers across the five CFIR domains for each respective program.

CFIR Domain	Kenya	Guyana	Türkiye	GMPELS
Intervention Characteristics	38%	50%	38%	38%
Outer Setting	75%	50%	100%	50%
Inner Setting	36%	29%	29%	21%
Characteristics of Individuals	50%	75%	75%	50%
Process	44%	44%	67%	33%

This is expressed as a percentage of the total barriers identified within each domain.

**Figure 1:**
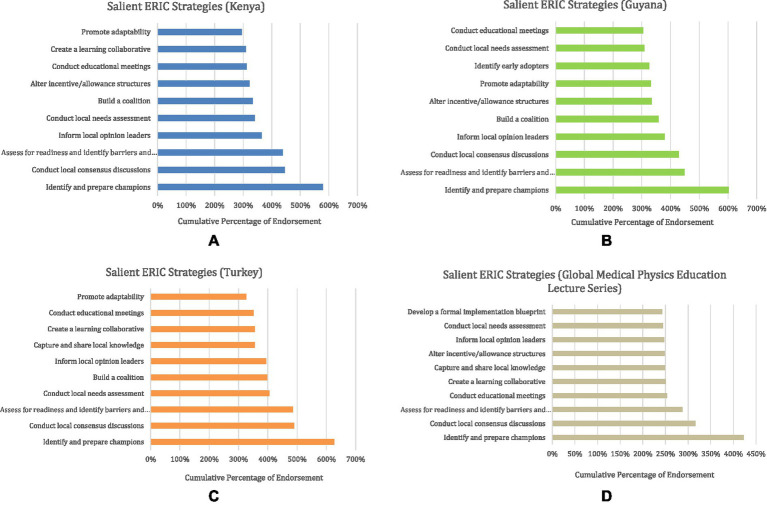
**(A–D)** ERIC strategies with the highest match value to the combination of identified barriers. Cumulative percentages represent the sum of expert endorsements across all selected CFIR barriers.

The program-specific strategies are shown in Figures 1A–D. Across the four programs, several recurring implementation challenges were identified by stakeholders for each respective initiative. Strategies to address these barriers provided by the CFIR-ERIC Matching Tool are also visualized in Figures 1A–D. The ERIC strategies with the highest cumulative percentage match value for each intervention are displayed. The cumulative percentages exceed 100% because implementation experts of the CFIR-ERIC tool endorse the same strategy for multiple barriers independently. Notably, barriers related to external technical support, costs, or policy constraints generated the lowest cumulative percentage match values, reflecting the strong collaborative and logistical groundwork of these initiatives.

Tables 3–6 presents the most salient implementation barriers identified in each respective program, as organized by the CFIR construct. Each barrier is paired with an ERIC strategy that is most strongly endorsed by expert respondents (*n* = 169) as effective for addressing that specific barrier. The percentages reflect the number of experts selecting the strategy among their top seven for an indicated barrier. The suggested ERIC strategy output is dependent on the specific combination of topics indicated by the stakeholders from each initiative.

**Table 3:** Top expert-endorsed ERIC strategies matched to CFIR-identified barriers in Kenya.

CFIR construct	Barrier description	Matched ERIC strategy	% experts endorsing strategy for barrier
Readiness for implementation	There are a few tangible and immediate indicators of organizational readiness and commitment to implement the innovation.	Assess for readiness and identify barriers and facilitators	81%
Access to knowledge and information	Stakeholders do not believe that the innovation can be sufficiently adapted, tailored, or reinvented to meet local needs.	Conduct educational meetings	79%
Patient needs and resources	Patient needs, including barriers and facilitators to meet those needs, are not accurately known, and/or this information is not a high priority for the organization.	Obtain and use patients/consumers and family feedback	76%
Adaptability	Stakeholders do not believe that the innovation can be sufficiently adapted, tailored, or reinvented to meet local needs.	Promote adaptability	73%
Patient needs and resources	Patient needs, including barriers and facilitators to meet those needs, are not accurately known and/or this information is not a high priority for the organization.	Involve patients/consumers and family members	71%

**Table 4:** Top expert-endorsed ERIC strategies matched to CFIR-identified barriers in Guyana.

CFIR construct	Barrier description	Matched ERIC strategy	% experts endorsing strategy for barrier
Readiness for implementation	There are a few tangible and immediate indicators of organizational readiness and commitment to implement the innovation.	Assess for readiness and identify barriers and facilitators	81%
Access to knowledge and information	Stakeholders do not have adequate access to digestible information and knowledge about the innovation, nor do they know how to incorporate it into work tasks.	Conduct educational meetings	79%
Adaptability	Stakeholders do not believe that the innovation can be sufficiently adapted, tailored, or reinvented to meet local needs.	Promote adaptability	73%
Champions	Individuals acting as champions who support, market, or ‘drive through’ implementation in a way that helps to overcome indifference or resistance from key stakeholders who are not involved or supportive.	Identify and prepare champions	67%
Cosmopolitanism	The organization is not well networked with external organizations.	Build a coalition	62%

**Table 5:** Top expert-endorsed ERIC strategies matched to CFIR-identified barriers in Turkish MRI course.

CFIR construct	Barrier description	Matched ERIC strategy	% experts endorsing strategy for barrier
Access to knowledge and information	Stakeholders do not have adequate access to digestible information and knowledge about the innovation, nor how to incorporate it into work tasks.	Conduct educational meetings	79%
Patient needs and resources	Patient needs, including barriers and facilitators to meet those needs, are not accurately known, and/or this information is not a high priority for the organization.	Obtain and use patients/consumers and family feedback	76%
Adaptability	Stakeholders do not believe that the innovation can be sufficiently adapted, tailored, or reinvented to meet local needs.	Promote adaptability	73%
Planning	A scheme or sequence of tasks necessary to implement the intervention has not been developed, or the quality is poor.	Develop a formal implementation blueprint	73%
Patient needs and resources	Patient needs, including barriers and facilitators to meet those needs, are not accurately known, and/or this information is not a high priority for the organization.	Involve patients/consumers and family members	71%

**Table 6:** Top expert-endorsed ERIC strategies matched to CFIR-identified barriers in the global medical physics education lecture series (GMPELS).

CFIR construct	Barrier description	Matched ERIC strategy	% experts endorsing strategy for barrier
Access to knowledge and information	Stakeholders do not have adequate access to digestible information and knowledge about the innovation, nor how to incorporate it into work tasks.	Conduct educational meetings	79%
Adaptability	Stakeholders do not believe that the innovation can be sufficiently adapted, tailored, or reinvented to meet local needs.	Promote adaptability	73%
Planning	A scheme or sequence of tasks necessary to implement the intervention has not been developed or the quality is poor.	Develop a formal implementation blueprint	73%
Key stakeholders	Multi-faceted strategies to attract and involve key stakeholders in implementing or using the innovation (e.g., through social marketing, education, role modeling, and training) are ineffective or non-existent.	Identify and prepare champions	63%
Goals and feedback	Goals are not clearly communicated or acted upon, nor do stakeholders receive feedback that is aligned with goals.	Audit and provide feedback	61%

**Figure 2:**
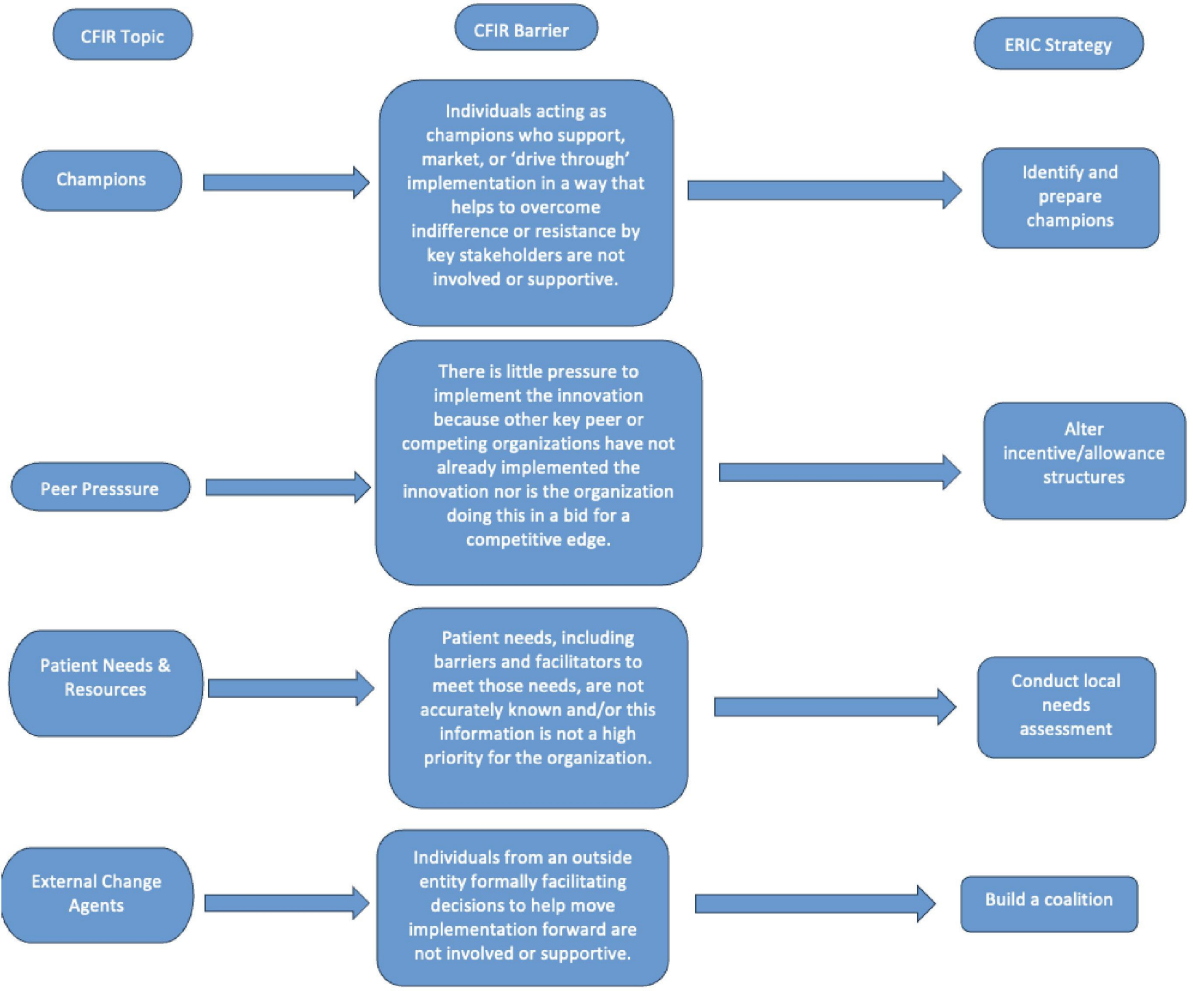
Process flowchart of salient CFIR barriers matched to corresponding ERIC strategy.

One prominent construct was *Adaptability*, the ability to tailor educational interventions to fit local needs and resources. For example, in Kenya, while the radiation oncology residents appreciated the didactic sessions, some of the material focused on advanced radiotherapy technologies, such as stereotactic radiosurgery, that were not available in their clinical setting. This technological mismatch occasionally limited the perceived immediate applicability of the content, suggesting opportunities to enhance contextual tailoring in future iterations. The construct *Planning* also appeared in both Kenya and Guyana, where it was recommended to be addressed with *Develop a Formal Implementation Blueprint* (73%), reflecting a shared gap in the formalized implementation process (Tables 3, 4; Figure 2).

Another strategy that commonly emerged was the importance of identifying and preparing local champions or individuals who supported the intervention to help sustain momentum. In Guyana, there has historically been limited integration of physics expertise into the residents’ education, as there are only two medical physicists present within the health system as of June 2024. Strengthening the alignment between physics leadership and clinical training could help amplify the long-term impact of educational efforts. *Identify and Prepare Champions* also appeared in Türkiye under a slightly different construct (Champions), reinforcing the broader relevance of internal leadership development.

The limited role of *Peer Pressure* was also noted, particularly in Türkiye, where the MRI course was among the first of its kind. Of the four total stakeholders, three identified *Peer Pressure* as a barrier within the CFIR Outer Setting. Within the CFIR framework, *Peer Pressure* refers to the pressure exerted by external organizations having already implemented said intervention. Without comparable local or regional programs to serve as benchmarks or sources of professional momentum, there was relatively less institutional incentive to formalize or expand the course beyond its initial offering. Addressing this may require building professional networks or creating institutional recognition to reinforce the perceived value of such an initiative.

While individual programs presented unique implementation contexts, several core strategies recurred across sites, including promoting adaptability through the development of locally relevant case materials and strengthening leadership capacity by supporting local champions. This convergence suggests that, although tailoring to context remains essential, there is value in leveraging shared evidence-based strategies to address common challenges in an underemphasized medical subspeciality.

## Discussion

This study offers insight into the implementation of four virtual educational initiatives designed to strengthen radiological capacity in LMIC settings. While each program operated within a distinct institutional and geographic context, several recurring barriers and corresponding strategies emerged. One of the most prominent constructs was *Adaptability*, which underscores the importance of aligning educational content with local clinical practice and technological availability. In Kenya, for example, the inclusion of advanced radiotherapy techniques, such as stereotactic radiosurgery, was perceived as less relevant, given that such technologies were not yet in use. Matching content to local resources may improve engagement and applicability, and the strategy “Promote Adaptability” was consistently endorsed for this barrier.

Another recurring barrier was planning. Both Kenya and Türkiye reported limited use of structured protocols to guide implementation. This gap was addressed through the ERIC strategy “Develop a Formal Implementation Blueprint,” which provides a framework for sequencing tasks, defining roles, and clarifying program goals. The absence of formal planning may undermine continuity and limit scalability, particularly for resource-constrained institutions seeking to replicate or sustain such programs.

Local leadership also played a significant role in influencing implementation outcomes. In Guyana, the limited integration of physics instruction into radiology residency training reflected both a structural gap and a lack of internal advocacy. At the time of the initiative, only two medical physicists were employed nationally, which constrained the visibility and influence of the discipline. This led stakeholders to identify “Champions” as a barrier, which matched the strategy “Identify and Prepare Champions.” In the CFIR framework, champions are individuals who actively promote and drive implementation, helping to overcome resistance and maintain momentum. In the context of medical physics, champions may serve as advocates for curricular inclusion, faculty support, or cross-disciplinary collaboration.

The absence of external reference points also emerged as a barrier in Türkiye. The construct “Peer Pressure” was identified by most stakeholders in reference to the MRI course, which was among the first of its kind in the country. In the absence of comparable programs to benchmark against, the course lacked institutional incentives for formalization or expansion. While CFIR defines peer pressure in a neutral sense, as the influence of external organizations implementing similar innovations, it may be useful to frame this concept more broadly as stakeholder alignment or sectoral signaling. Strategies such as building coalitions with professional societies or linking courses to certification pathways may help address this type of barrier.

While the primary focus of this study was identifying barriers, several supporting factors also became apparent. In the GMPELS initiative, for instance, the program evolved over time in response to participant feedback. Early sessions were sometimes misaligned with learner expectations, but adjustments were made to include topics such as proton therapy and MRI-guided radiotherapy (17). This iterative responsiveness strengthened the program’s reach and relevance. Similar adaptations may improve sustainability across settings.

Several limitations should be acknowledged. First, data were collected retrospectively and relied on stakeholder recall, which may introduce bias. Future studies could reduce this limitation through prospective data collection or triangulation with participant-level outcomes. Second, although the stakeholder sample represented all individuals directly involved in implementation, the small number of respondents limits the generalizability of findings. Third, while CFIR offers a useful framework for categorizing implementation factors, it does not completely account for broader structural determinants such as national workforce shortages, health financing, or regulatory frameworks. Recent proposals to expand CFIR with a sixth domain, *Characteristics of Systems,* may better capture these macro-level influences (18).

Despite these constraints, this study demonstrates how structured implementation frameworks can be applied to educational initiatives in radiological health. By linking CFIR constructs to targeted strategies, implementers can move beyond intuitive decision-making and adopt evidence-informed approaches that are responsive to local needs (22). The results suggest that, although contextual tailoring is essential, certain strategies, such as promoting adaptability, preparing local champions, and conducting structured educational meetings, may be broadly effective across settings.

## Conclusion

Virtual education offers a scalable means of strengthening the medical physics workforce in LMICs, but successful implementation depends on careful alignment with the local context. By applying the Consolidated Framework for Implementation Research and the ERIC Matching Tool, this study identified recurring barriers and mapped them to evidence-based strategies across four distinct programs. Despite differences in geography and institutional structure, constructs such as *Adaptability*, *Planning*, and *Access to knowledge* appeared consistently, along with strategies that emphasized educational meetings, local leadership, and structured implementation planning. These findings support the utility of implementation science frameworks in designing and sustaining global health education efforts and point to a core set of strategies that may improve uptake, engagement, and long-term impact. Future research should continue to refine these approaches through prospective evaluation and integration with broader health system strengthening initiatives.

## Data Availability

The raw data supporting the conclusions of this article will be made available by the authors, without undue reservation.
